# Dual targeting of NUAK1 and ULK1 using the multitargeted inhibitor MRT68921 exerts potent antitumor activities

**DOI:** 10.1038/s41419-020-02885-0

**Published:** 2020-09-01

**Authors:** Yiran Chen, Xiaoling Xie, Chunsheng Wang, Yuxing Hu, Honghao Zhang, Lenghe Zhang, Sanfang Tu, Yanjie He, Yuhua Li

**Affiliations:** 1 Department of Hematology, Zhujiang Hospital, Southern Medical University, Guangzhou, China; 2 Bioland Laboratory (Guangzhou Regenerative Medicine and Health Guangdong Laboratory), Guangzhou, China

**Keywords:** Autophagy, Target validation, Cell death and immune response, Cancer immunotherapy, Drug development

## Abstract

Utilizing oxidative stress has recently been regarded as a potential strategy for tumor therapy. The NUAK family SNF1-like kinase 1 (NUAK1) is a critical component of the antioxidant defense system and is necessary for the survival of tumors. Therefore, NUAK1 is considered an attractive therapeutic target in cancer. However, antioxidant therapy induced elevated ROS levels to activate the Unc-51-like kinase 1 (ULK1) pathway to promote protective autophagy and ULK1-dependent mitophagy. Thus, the combined inhibition of NUAK1 and ULK1 showed a strong synergistic effect in different tumor types. Herein, the potential antitumor activities of a dual NUAK1/ULK1 inhibitor MRT68921 were evaluated in both tumor cell lines and animal models. MRT68921 significantly kills tumor cells by breaking the balance of oxidative stress signals. These results highlight the potential of MRT68921 as an effective agent for tumor therapy.

## Introduction

Tumor cells possess an infinite drive to proliferate, exposing them to more severe metabolic stress than normal cells^1^. Increased metabolic stress promotes the production of elevated reactive oxygen species (ROS), influencing downstream signals and inducing cell death. The characteristics of tumor cells make them more sensitive to changes in oxidative stress, which is the mechanism of several anticancer therapies^2^. As excessive ROS promote lethal oxidative stress and damage cells, the protective functions of the antioxidant defense system are critical for tumor survival under stress. Recently, targeting the antioxidant defense system of tumors has been considered as a potentially effective strategy for tumor therapy^3,4^.

NUAK1 (also known as ARK5) is one of 12 kinases from the AMPK subfamily and is critical for maintaining metabolic homeostasis by regulating the mitochondrial respiratory chain complex and the metabolism of glutamine^5^. Many studies have reported that NUAK1 is critical for the survival of cancer patients. Elevated NUAK1 expression in different cancer types represents worse malignant behaviors, including chemotherapeutic resistance, early-stage metastasis, and poorer outcome^5–9^. These results suggest that NUAK1 is a protective factor for cancer cells in disease development and progression via mechanisms of epithelial–mesenchymal transition (EMT) and metabolic regulation^10^. Recently, several important studies have highlighted that NUAK1, as a key component of the antioxidant response pathway, is associated with aggressive disease and poor outcome in cancer patients through suppressing Gsk3β-dependent inhibition of NRF2 nuclear localization^11,12^. Depletion of NUAK1 by siRNA or small molecule inhibitors prolongs survival in mouse models of different tumors, demonstrating that targeting cellular energy homeostasis by inhibiting NUAK1 is a valid therapeutic strategy^12^.

However, the effectiveness of NUAK1-targeting therapies is still unsatisfactory^13^, which may be due to the inherent defense mechanisms of tumors, such as DNA damage repair, negative feedback, bypass signals, or autophagy^14^. Autophagy is an evolutionarily conserved intracellular catabolic process that is upregulated under conditions of perceived stress and in response to cellular damage^15^. Evidence has proven that autophagy is a protective effect in response to lethal oxidative stress^16^. ULK1 is the autophagy initiator and is the only serine-threonine kinase in mammals^17^. Accumulating evidence suggests that ULK1 is a multifunctional target for potential therapeutic applications^18^. Blocking the early stage of autophagy by ULK1 inhibition significantly potentiates chemosensitivity, and these effects are superior to late-stage inhibition by chloroquine^19^. Besides the critical role in autophagy flux, ULK1 is required for targeting of mitochondria and to lysosomes in mitophagy process^20^. ULK1 could translocate to mitochondria and phosphorylate FUNDC1 to regulate mitophagy^21^. Therefore, we predict that dual inhibition of NUAK1 and ULK1 could induce a significant synergistic cytotoxic effect on various cancer types.

In our study, we sought to determine whether selectively inhibiting NUAK1 and ULK1 could be an effective way to target oxidative stress homeostasis in cancer cells. Our findings demonstrate a synergistic anticancer effect in response to combined treatment with NUAK1 inhibitor (WZ4003) and ULK1 inhibitor (SBI-0206965) in different types of cancer cells. Our study demonstrates a significant anticancer effect in response to MRT68921, a dual NUAK1/ULK1 inhibitor^22,23^, and MRT68921 has a strong cytotoxic effect on different cancer cell lines and animal models while sparing normal cells. Our study also suggests that MRT68921 has the potential to inhibit cancer metastasis. To further analyze the binding mode between MRT68921 and NUAK1, we established a homology model of the NUAK1 kinase and performed molecular docking. In summary, our study has demonstrated a new therapeutic strategy for inhibiting cancer growth with dual-targeting antioxidant mechanisms and mitophagy using a NUAK1/ULK1 dual inhibitor, MRT68921.

## Materials and methods

### Cell lines, culture conditions, and chemicals

The human cancer cell lines A549, H1299, NCI-H460, MNK45, U251, SW480, SW620, HCT116, Colo320 and HT-29, PC-3, U266, and the mouse breast cancer cell line 4T1 were cryopreserved in the Hematological Laboratory of Zhujiang Hospital (Guangzhou, China). All cell lines were incubated in DMEM medium supplemented with 10% fetal bovine serum at 37 °C with 5% CO_2_. WZ4003, SBI-0206965, Chloroquine, and MRT68921 were purchased from Selleckchem (Houston, TX, USA), dissolved in DMSO or water, and stored at −20 °C. CCK-8 was purchased from Dojindo Laboratories (Japan). Mitotracker, DAPI, and TritonX-100 were purchased from Solarbio (Beijing, China).

### Transient transfection

For transfections, three siRNAs against human NUAK1 (siNUAK1–1: TCC TGA AGA AGC GAA GCA A; siNUAK1–2: TCG ATG ACA ACT GCA ATA T; and siNUAK1–3: AGA GAG AAT CAG GTT ACT A) and a scrambled siRNA were synthesized by RiboBio (Guangzhou, China).

### Colony formation

The cells were seeded at a density of 400 cells per well in 6-well plates and cultured overnight before treatment with DMSO or MRT68921 for 24 h. The cells were then washed once with PBS and returned to full growth medium for 14 days. Cells were stained with 0.1% crystal violet after fixation with 100% methanol.

### Microscopy and flow cytometry

To examine the morphology of apoptotic and necrotic cells, the cells were seeded in 6-well plates at ~70% confluency and subjected to the indicated treatments. For flow cytometry analyses, cells were treated with various chemotherapy drugs as indicated. Cells were harvested, washed with PBS three times, and stained using Annexin V-FITC/PI Apoptosis Assay Kit (BD Pharmingen, San Jose, CA, USA) and DCFH-DA Reactive Oxygen Species Assay Kit (Meilunbio, China) according to the manufacturer’s instructions. Stained cells were analyzed with a BD FACS flow cytometer (BD Biosciences, Franklin Lakes, NJ), and the data were processed using FlowJo software.

### Western blot analysis

All prepared cells were homogenized in protein lysate buffer. The protein concentrations were determined using a Bradford protein assay kit (Beyotime, China). After addition of the loading buffer, protein samples were electrophoresed, transferred to PVDF membranes (Millipore, Billerica, MA, USA), and subsequently blocked. The membranes were immunoblotted with rabbit anti-human primary antibody overnight at 4 °C. Antibodies against NUAK1, ULK1 (#8054), p-ULK1 (Ser317, #37762), LC3A/B (#4108), MYPT1 (#2634), p-MYPT1 (Ser668, #3048), ATG13 (#13273), p-ATG13 (Ser355, #46329), Gsk3β (#9832), and p-Gsk3β (Ser9, #9336) were obtained from Cell Signaling Technology (Danvers, MA, USA). Antibodies against PARP1 (#13371-1-AP), p62/SQSTM1 (#18420-1-AP), and GAPDH (#60004-1-Ig) were obtained from Proteintech (Chicago, USA). After three washes with TBST, the blots were incubated with horseradish peroxidase (HRP)-conjugated secondary antibodies for 1 h, and the HRP signal was detected using an enhanced chemiluminescence reagent (Pierce Biotechnology, Rockford, IL, USA).

### Wound healing and transwell assays

A549, Colo320, and U251 cells were seeded in 6-well plates at a density of 5 × 10^5^ cells per well. Wounds were made when cells were over 90% confluent. Cells were treated with MRT68921 or DMSO for 48 h. The wounds were photographed at 0, 24, and 48 h. For transwell assays, NCI-H460 and U251 cells were suspended in serum-free medium and seeded into the upper chambers of the transwell insert. The lower chambers were filled with medium containing 20% FBS. DMSO and MRT68921 were added into the upper chambers the following day. After 12 h of incubation, the migrated cells on the bottom chambers were fixed with 4% paraformaldehyde and stained with crystal violet. The number of migrated cells was counted under a bright-field microscope.

### Immunofluorescence staining and co-localization analysis

Cells were suspended in DMEM with 10% FBS and seeded in 24-well plate with coverslips. After overnight culture, cells were treated with corresponding drugs (WZ4003, SBI-0206965, chloroquine, MRT68921, or combinations) for different time. After treatment, cells were fixed by paraformaldehyde and treated with 5% TritonX-100 for 20 mins. After confined with 2% BSA, cells were incubated with fluorescent antibodies (ULK1: Abcam, #ab240916, 1:50; LC3B: CST, 1:50) overnight. Then, cells were cultured with secondary antibody (Alexa Fluor 488-Goat Anti-Rabbit IgG(H + L), Proteintech, 1:100) for 2 h in dark. After that, cells were treated with mitotracker and DAPI in turn. Coverslips were upside-down on slides with a sealing agent and observed under immunofluorescence microscope or laser scanning confocal microscope.

### In vivo tumor models

All animal studies were carried out in accordance with Southern Medical University’s Policy on Care and Use of Laboratory Animals. Five-week-old female BALB/c nude mice and BALB/c mice were purchased from Guangdong Medical Laboratory Animal Center. Animals were housed at constant room temperature with a 12 h light/12 h dark cycle and fed a standard rodent diet and water. NCI-H460 and MNK45 cells were harvested and injected subcutaneously (5 × 10^6^ cells in 200 μL of PBS) into nude mice. The treatments started after the size of the tumors reached 50 mm^3^, mice with low size (<50 mm^3^) of tumors or large size (>100mm^3^) of tumors were excluded. Included mice were randomly and blindly divided into different groups. NCI-H460-injected mice were peritumorally and subcutaneously injected with DMSO or MRT68921 (10, 20, or 40 mg/kg/d) every day until the seventh treatment. MNK45-injected mice were peritumorally and subcutaneously injected with DMSO or MRT68921 (20 mg/kg/d) every 2 days until the seventh treatment. The tumor volumes were assessed every 3 days and calculated using the following equation: (length × width^2^)/2. Mice were killed when the tumor volume exceeded 3000 mm^3^; at which point, the tumor samples were excised, fixed, and embedded in paraffin for immunohistochemical analyses. 4T1 cells were harvested and injected intravenously (2 × 10^5^ cells in 100 μL of PBS) into BALB/c mice. The treatment started on the third day after injection. All mice were randomly and blindly divided into different groups. The mice were intravenously injected with DMSO or MRT68921 (20 mg/kg/d) every day until the seventh treatment. The Kaplan–Meier method was used to measure overall survival. Lung tissues were excised, fixed, and stained by H&E for the counting of metastatic nodes.

### Homology modeling

The target sequence of NUAK1 was acquired from UniProt with the UniProt ID of O60285. Template crystal structures were identified through BLAST and downloaded from RCSB Protein Data Bank (PDB ID: 5ES1). Homology modeling was conducted in MOE^24^. The protonation state of the protein and the orientation of the hydrogens were optimized by LigX at a pH of 7 and a temperature of 300 K. First, the target sequence was aligned to the template sequence, and ten independent intermediate models were built. Then, the intermediate model that scored best according to the GB/VI scoring function was chosen as the final model, subject to further energy minimization using the AMBER10: EHT force field.

### Molecular docking

The 2D structures of molecules MRT68921, WZ4003, and HTH-01-015 were drawn in ChemBioDraw 2014 and converted to 3D structures in MOE through energy minimization. Prior to docking, the force field of AMBER10: EHT and the implicit solvation model of Reaction Field (R-field) were selected. MOE-Dock was used for molecular docking simulations of molecules with NUAK1. The docking workflow followed the “induced fit” protocol, in which the side chains of the receptor pocket were allowed to move according to ligand conformations, with a constraint on their positions. The weight used for tethering the sidechain atoms to their original positions was 10. For each ligand, all docked poses were ranked by London dG scoring first, then a force field refinement was carried out on the top 20 poses followed by a rescoring of GBVI/WSA dG. The conformations with the lowest free energies of binding were selected as the best (probable) binding modes. Molecular graphics were generated by PyMOL.

### Statistical analysis

Statistical significance was evaluated using SPSS 11.0 and GraphPad Prism 7. The quantitative data are presented as the mean ± standard deviation (SD). Statistical differences between the groups were analyzed with Student’s *t*-test. *P* < 0.05 was considered statistically significant. * represents *P* < 0.05, ** represents *P* < 0.01, and *** represents *P* < 0.001.

## Results

### Inhibition of NUAK1 kinase activity-induced autophagy in cancer cells

**Fig. 1:**
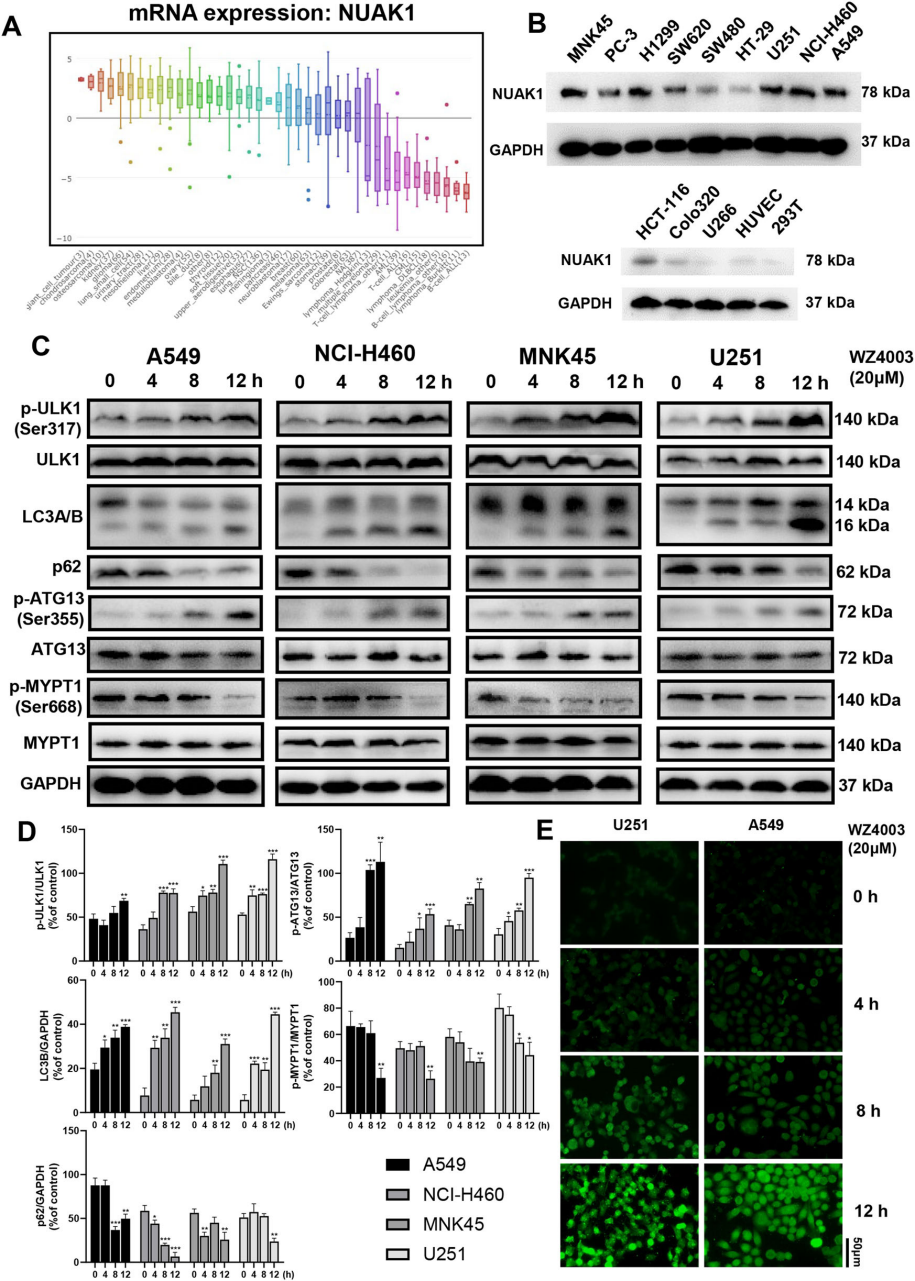
NUAK1 is overexpressed in different types of cancer cells, and NUAK1 inhibition induces ULK1-dependent autophagy formation. **a** Expression levels of NUAK1 were screened in the Broad Institute Cancer Cell Line Encyclopedia (CCLE). **b** NUAK1 expression levels were analyzed by western blot in 12 cancer cell lines and 2 normal cell lines. **c** A549, NCI-H460, MNK45, and U251 cells were treated with 20 μM NUAK1 inhibitor WZ4003 for 0, 4, 8, and 12 h and analyzed by western blot. WZ4003 treatment induces phosphorylation of ULK1 and puncta of LC3 together with downregulation of p62 and upregulation of phosphorylated ATG13. Phosphorylation of NUAK1 downstream target MYPT1 is downregulated by WZ4003. **d** Phosphorylated ULK1 protein levels (normalized to total ULK1), phosphorylated ATG13 protein levels (normalized to total ATG13), phosphorylated MYPT1 protein levels (normalized to total MYPT1), p62 protein levels (normalized to GAPDH), and LC3B protein levels (normalized to GAPDH), *n* = 3. **e** Representative images of LC3B fluorescence in U251 cells and A549 cells treated with 20 μM WZ4003 for 0–12 h.

Previous studies and data in CCLE (Cancer Cell Line Encyclopedia, Fig. 1a) suggest that NUAK1 is overexpressed in many solid tumor cell lines. We also observed elevated NUAK1 expression in different tumor types, while the expression of NUAK1 was slight in normal cell lines (Fig. 1b). NUAK1 has a critical role in the adaptive antioxidant response, which is associated with aggressive cancer and worse outcome. Oxidative stress-induced ROS production damages mitochondria, which in turn phosphorylates ULK1, leading to the recruitment of mitochondria and initiation of mitophagy. Therefore, ULK1 activity may contribute to the survival of tumor cells under the conditions of NUAK1 inhibition and oxidative stress. Autophagy was evaluated by analysis of LC3A/B, p-ATG13/ATG13, and p62 expression. A time-dependent increase in LCB expression and phosphorylation of ATG13 combined with a decrease in p62 expression was observed in A549, NCI-H460, MNK45, and U251 cells after WZ4003 treatment. WZ4003 treatment also increased the levels of phosphorylated ULK1 and decreased the phosphorylation of MYPT1, a downstream target of NUAK1 (Fig. 1c, d). U251 cells were treated with siRNA for 48 h to knockdown NUAK1 expression, subsequent western blotting suggested increased LC3B expression together with decreased p62 expression (Supplementary Fig. S1B, C). The induction of autophagy was also checked by LC3B expression by Immunofluorescence staining. WZ4003 significantly enhance fluorescent LC3B in U251 and A549 cells in a time-dependent manner (Fig. 1e).

### Combination effects of WZ4003 and ULK1 inhibitors

**Fig. 2:**
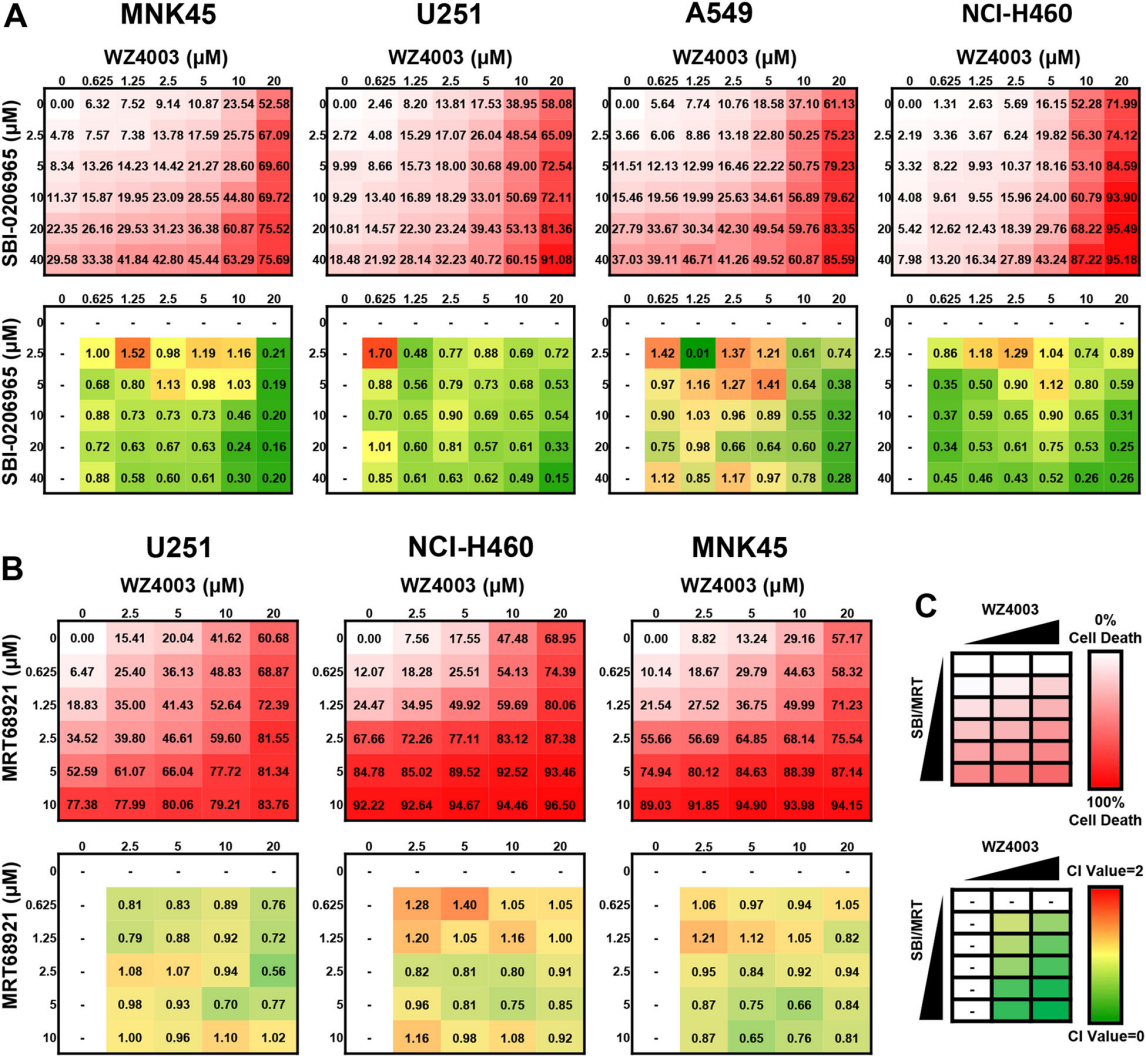
The NUAK1 inhibitor WZ4003 exhibits broad synergy with the ULK1 inhibitor SBI-0206965 in NUAK1-overexpressing cancer cell lines. **a** The average cell death and CI value quantification for the 7 × 6 matrices of WZ4003 and SBI-0206965 in MNK45, U251, A549, and NCI-H460 cells. **b** The results of average cell death induced by WZ4003 + MRT68921 combinations evaluated in the 5 × 6 matrices and corresponding quantification of synergy CI values. **c** A schematic of the combination treatment of WZ4003 plus reported ULK1 inhibitors. The CI value was calculated for cell death induced by each combination group. CI combination index.

Because WZ4003 activated autophagy through a ULK1-dependent mechanism, we considered that the addition of ULK1 inhibitors could inhibit this protective effect and enhance WZ4003 activity. SBI-206965^25^ is a highly selective kinase ULK1 inhibitor with an IC50 of 108 nM, and MRT68921^23^ is a dual autophagy kinase inhibitor of ULK1 and ULK2 with IC50 values of 2.9 and 1.1 nM, respectively. The ability of WZ4003 to induce cell death in combination with ULK1 inhibitors was assessed in different cancer cell lines. A 6 × 7 matrix was designed that contained six WZ4003 concentrations and five SBI-0206965 concentrations for 24-h treatments. All cells were seeded into 96-well plates and proliferated into 80% confluence before treatment. Then the percentages of cell death were measured by CCK-8 assay (Fig. 2c). The Chou–Talalay method^26^ was used to quantify the effects, and the combination index (CI) values were calculated for each combination (Fig. 2c). Four cancer cell lines (MNK45, U251, A549, and NCI-H460) were used for these experiments, and all four cell lines treated with combinations of WZ4003 and SBI-0206965 demonstrated significantly enhanced cell death at multiple concentrations in the 6 × 7 matrix (Fig. 2a). Strong synergy was observed when the concentration of WZ4003 exceeded 20 μM.

**Fig. 3:**
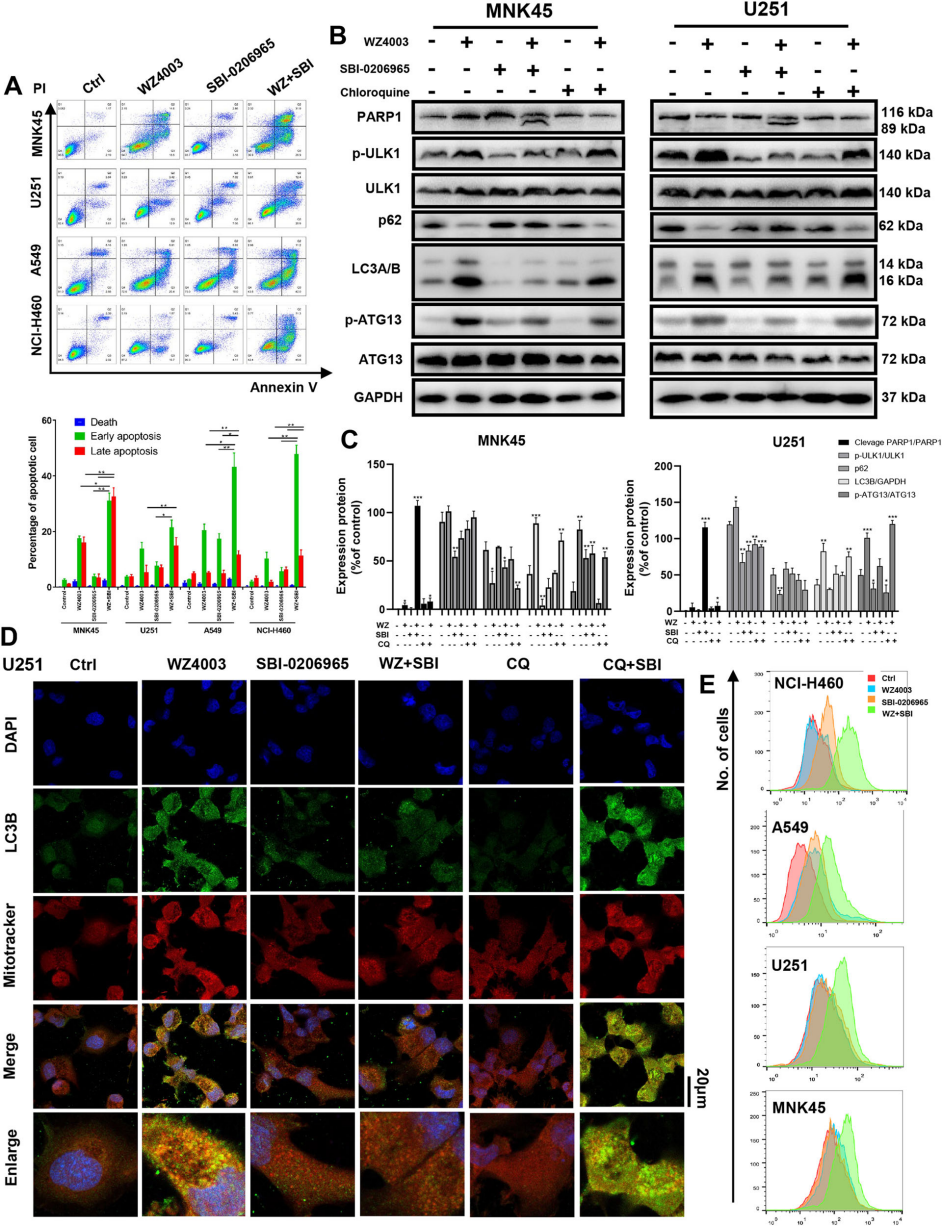
ULK1 inhibitor SBI-0206965 synergizes with WZ4003 by inhibiting NUAK1 inhibition-induced mitophagy. The cancer cell lines MNK45, U251, A549, and NCI-H460 were treated with WZ4003 (20 μM), SBI-0206965 (20 μM) or a combination of WZ4003 plus SBI-0206965, chloroquine (CQ, 20 μM) was set as controls. **a** The induction of apoptosis was analyzed by Annexin V-FITC and propidium iodide staining after 12 h of treatment. **b** Western blot analysis for p-ULK1/ULK1, p-ATG13/ATG13, cleavage of apoptosis marker PARP1, LC3A/B, and p62 after 8 h of treatment. **c** Quantifications of P-ULK1/ULK1, p-ATG13/ATG13, p-MYPT1/MYPT1, p62/GAPDH, and LC3B/GAPDH (*n* = 3). **d** Representative and merge images showing LC3B fluorescence (green), mitotracker fluorescence (red), and DAPI fluorescence (blue) in U251 cell after treatment of WZ4003 (20 μM), SBI-0206965 (20 μM), CQ (20 μM), or combinations for 8 h. Enlarged merge images representative the co-localizations of LC3B-labeled autophagosome and mitotracker-labeled mitochondria. **e** ROS levels were analyzed by DCFH-DA staining after 8 h of treatment. Combination treatment induced lethal enhancement of ROS levels. ROS reactive oxygen species.

The synergistic effects of cell death induced by the WZ4003/SBI-0206965 combination were further assessed for apoptosis and ROS levels by flow cytometry (Fig. 3a, e) and for cleavage of PARP1 by western blot (Fig. 3b). Treatment of four cancer cell lines with the WZ4003/SBI-0206965 combination followed by Annexin V/PI staining revealed significant synergy, with the elevated improvement of Annexin V+/PI− or Annexin V+/PI+ cells, indicative of enhanced apoptosis (Fig. 3a). Combined treatment followed by DCFH-DA staining revealed a deficiency in the ROS balance induced by the WZ4003/SBI-0206965 combination (Fig. 3e). Morphology change observed under a microscope suggests significant cell death induced by treatment (Supplementary Fig. S2B). Western blot analysis of MNK45 and U251 revealed cleavage of PARP after combined treatment (Fig. 3b, c). The phosphorylation of ULK1 and ATG13 combined with puncta of LC3 increased after WZ4003 treatment and inhibited by adding SBI-0206965. WZ4003 combined with CQ also induces an increase of puncta LC3, which suggests WZ4003 could induce autophagy and this protective autophagy will be blocked by SBI-0206965 (Fig. 3b, c).

The combination of WZ4003 and MRT68921 was explored in U251, NCI-H460, and MNK45 (three different cancer types) (Fig. 2b). MRT68921, reported as a ULK1 inhibitor, blocks autophagosome initiation and potentiates chemosensitivity in mesothelioma. However, the combination of WZ4003 and MRT68921 did not show significant synergistic effects but did show additive effects in the 5 × 6 matrix (Fig. 2b). In particular, the significant cytotoxic effects of MRT688921 alone were observed in three cancer cell lines when its concentration exceeded 2.5 μM (Fig. 2b). The working concentrations of MRT68921 in previous studies were equal to or less than 1 μM, which showed almost no cytotoxic effect on neither cancer cells nor normal cells. Therefore, we suggest for the first time that MRT68921 has potential anticancer activity as a single-drug therapy.

### SBI-0206965 synergizes with WZ4003 by blocking protective mitophagy

WZ4003 treatment will enhance lethal oxidative stress, which may induce protective mitophagy. Previous researches have proved ULK1 is a critical protein in promote mitophagy^20,21^. Therefore, we propose that SBI-0206965 could enhance the cytotoxic effect of WZ4003 by blocking ULK1-induced protective mitophagy. We performed an immunofluorescence co-localization by confocal microscope to investigate whether p-ULK1 is required in oxidative stress-induced mitophagy under WZ4003 treatment. We found co-localization of p-ULK1 and mitochondria after WZ4003 treatment in U251 cells (Supplementary Fig. S1A). Then, we performed a co-localization of autophagosome stained by LC3B and mitochondria stained by mitotracker under laser scanning confocal microscope (Fig. 3d). A typical co-localization of LC3B and mitotracker was observed after WZ4003 treatment. WZ4003 combined with SBI-0206965 induced a failure of co-localization and a decrease of LC3B expression. As a control, WZ4003 combined with chloroquine induce an extremely expressed LC3B (Fig. 3d). These results suggest SBI-0206965 could inhibit NUAK1-induced autophagy and mitophagy, which will enhance the cytotoxic effect of WZ4003.

### Homology modeling of NUAK1 and molecular docking

**Fig. 4:**
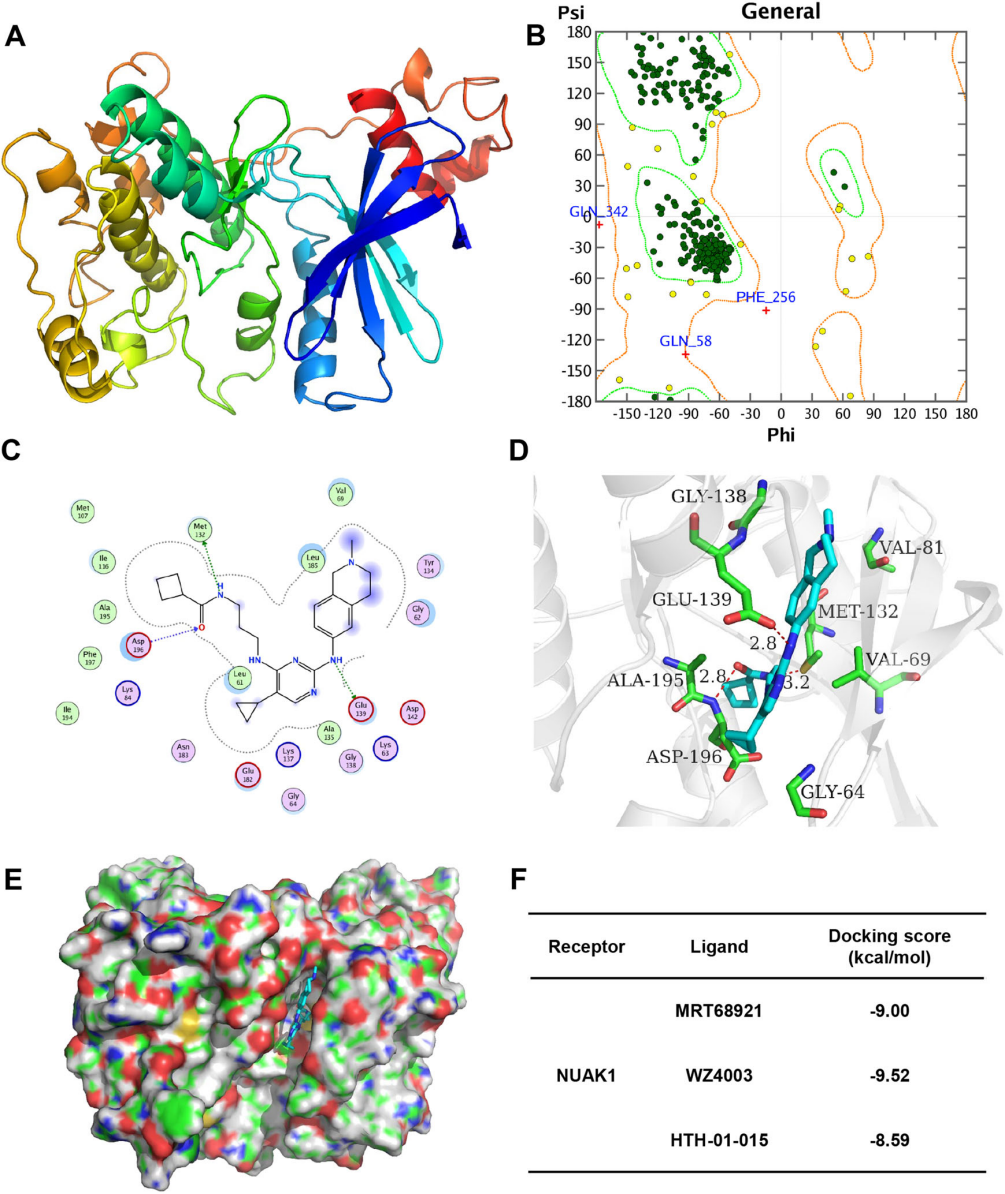
Homology modeling of NUAK1 and molecular docking. MRT68921 is a potential NUAK1 inhibitor. **a** The homology model of NUAK1. **b** Ramachandran plot for NUAK1. Dark green dots represent the residues in favored regions; yellow dots represent the residues in allowed regions, and the red cross represents the residues in irrational regions. **c** The 2D-binding mode of MRT68921 with NUAK1. **d** The 3D-binding mode of MRT68921 with NUAK1. **e** The surface-binding mode of MRT68921 with NUAK1. MRT68921 is colored in cyan, and the surrounding residues in the binding pockets are colored in green. The backbone of the receptor is depicted as a light blue cartoon. **f** The docking scores of MRT68921, WZ4003, and HTH-01-015 binding with human protein NUAK1.

A kinase profiling of MRT68921 performed in a previous study revealed that MRT68921 is a relatively specific ULK1 kinase inhibitor and a NUAK1 kinase inhibitor. Therefore, we performed homology modeling and molecular docking to analyze the potential of MRT68921 as a NUAK1/ULK1 dual inhibitor. The modeling result for NUAK1 is depicted in Fig. 4a, b. The Ramachandran plot for NUAK1 showed that more than 99% of residues are in allowed regions, indicating that the 3D structure of the model is reasonable. The structural analysis of the NUAK1 modeling results is shown in Supplementary Fig. S4A. The NUAK1 structure is basically consistent with the template structure. The average RMSD value of the three-dimensional structure overlap is 0.705 Å. The overall identity of the amino acid sequence was 49% (Supplementary Fig. S4B).

To investigate the binding mode of molecules with NUAK1, docking simulation studies were carried out. The NAUK1 docking scores of MRT68921, WZ4003, and HTH-01-015, which are −9.00, −9.52, and −8.59 kcal/mol, respectively, are shown in Fig. 4f. This computational result indicates that MRT68921, WZ4003, and HTH-01-015 can interact with NUAK1.

The binding mode of MRT68921 with NUAK1 is illustrated in Fig. 4c–e. The nitrogen atom of MRT68921, regarded as a hydrogen bond donor, forms one hydrogen bond with the oxygen atom of the carboxyl group of Glu139 of NUAK1. The oxygen atom of the amido group of MRT68921, regarded as a hydrogen bond acceptor, forms one hydrogen bond with the nitrogen atom of the backbone of Asp196 of NUAK1. The nitrogen atom of the amido group of MRT68921, regarded as a hydrogen bond donor, forms one hydrogen bond with the sulfur atom of the thioether group of Met132 of NUAK1. The binding mode of WZ4003 with NUAK1 is illustrated in Supplementary Fig. S4C. The oxygen atom of the amido group of WZ4003, regarded as a hydrogen bond acceptor, forms one hydrogen bond with the nitrogen atom of the backbone of Gly64 of NUAK1. The nitrogen atom of the amido group of WZ4003, regarded as a hydrogen bond donor, forms one hydrogen bond with the oxygen atom of the carboxyl group of Asp196 of NUAK1. The pyrimidine ring of WZ4003 forms a π-H conjugation with the carbon atom of the sidechain of Val69 of NUAK1. The binding mode of HTH-01-015 with NUAK1 is illustrated in Supplementary Fig. S4D. The pyrazole ring of HTH-01-015 forms a π-H conjugation with the carbon atom of the backbone of Gly138 of NUAK1. The pyrimidine ring of HTH-01-015 forms a π-H conjugation with the carbon atom of the sidechain of Val69 of NUAK1.

In summary, through docking simulation studies, we found that WZ4003 interacts with Gly64, Val69, and Asp196 of NUAK1 through π–H conjugation and hydrogen bond interactions. HTH-01-015 interacts with Val69 and Gly138 of NUAK1 through π–H conjugation and hydrogen bond interactions. The compounds WZ4003 and HTH-01-015 are known inhibitors of NUAK1, indicating that residues Gly64, Val69, Gly138, and Asp196 of NUAK1 may be important sites for inhibitor binding. The docking results showed that MRT68921 could form a hydrogen bond interaction or π–H conjugate with the residues Met132, Glu139, and Asp196 of NUAK1, indicating that MRT68921 may be a potential inhibitor of NUAK1.

### MRT68921 exhibits cytotoxic activity in cancer cells

**Fig. 5:**
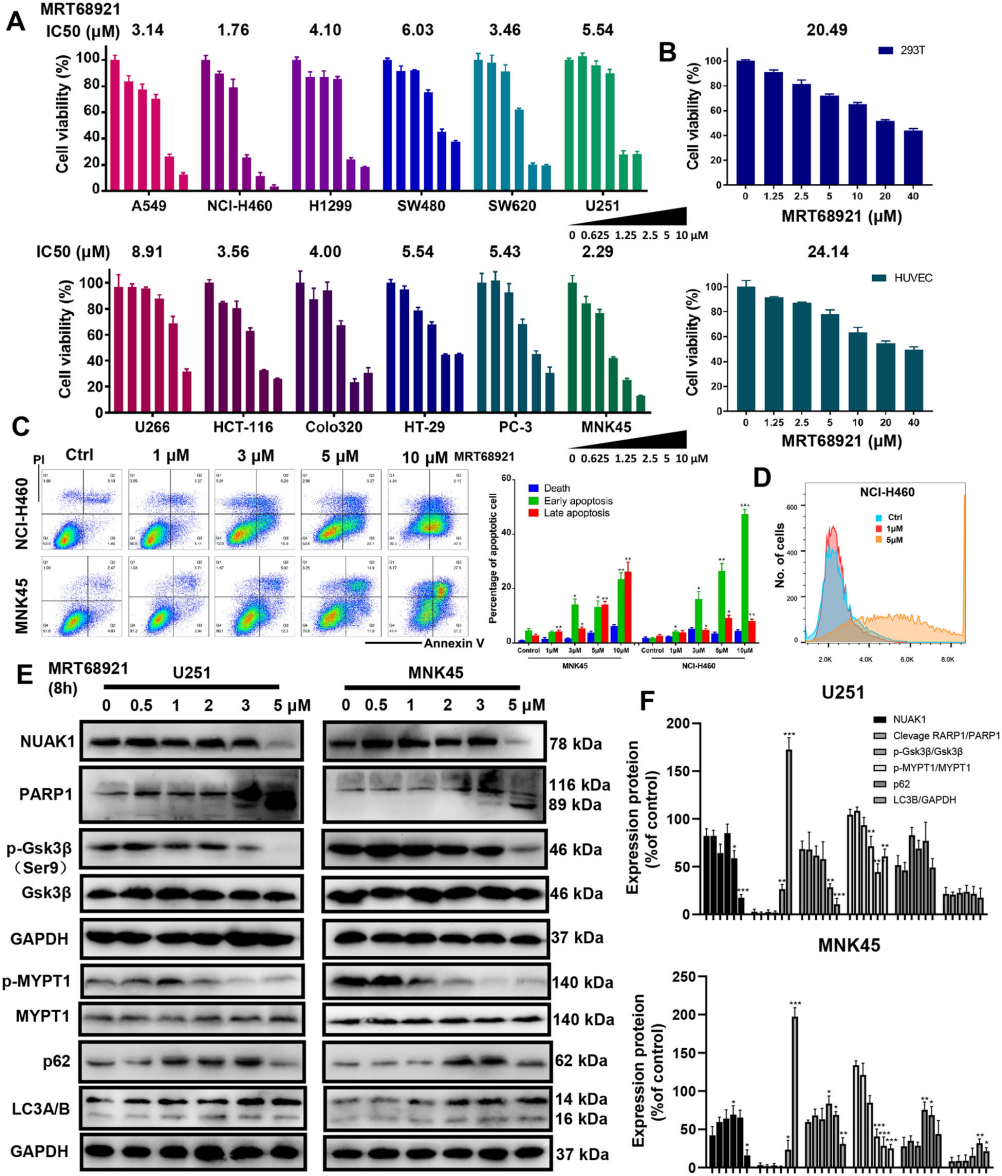
The dual NUAK1 and ULK1 inhibitor MRT68921 significantly induces apoptosis, elevates ROS levels, and inhibits the NUAK1/Gsk3β pathway in different cancer types. **a** Twelve cancer cell lines were treated with MRT68921 at different concentrations (from 0 to 10 μM) for 24 h, followed by analysis for cytotoxic effects with CCK-8 assay (*n* = 3). **b** 293 T and HUVECs were treated with MRT68921 (from 0 to 40 μM) for 24 h and evaluated by CCK-8 assay. MRT68921 has a significantly stronger cytotoxic effect on cancer cells than normal cells (*n* = 3). **c** NCI-H460 and MNK45 cells were treated with MRT68921 (from 0 to 10 μM) for 24 h, followed by apoptosis analysis with Annexin V/PI staining. A significant increase in both early and late apoptotic populations was observed. **d** NCI-H460 cells were treated with three different concentrations of MRT68921 (0, 1, and 5 μM) for 8 h, followed by ROS detection with DCFH-DA staining. Treatment with 5 μM MRT68921 induces elevated ROS levels. **e** U251 and MNK45 cells were treated with different concentrations of MRT68921 (from 0 to 5 μM) for 8 h, followed by western blot. **f**, Quantifications of cleavage PARP1/PARP1, p-Gsk3β/Gsk3β, p-MYPT1/MYPT1, NUAK1/GAPDH, p62/GAPDH, and LC3B/GAPDH (*n* = 3).

Considering the role of the NUAK1 and ULK1 pathways in cancer development, we evaluated the cytotoxic activity of MRT68921 in different cancer cell lines (A549, H1299, NCI-H460, et al.) and normal cell lines (293T & HUVEC). All cells were seeded in 96-well plates and proliferated into 80% confluence. All cells were treated with different concentrations of MRT68921 for 24 h and analyzed by CCK-8 assay. We observed that MRT68921 could significantly kill cancer cell lines with IC_50_ values ranging from 1.76 to 8.91 μM (Fig. 5a). NCI-H460 cells were the most sensitive to MRT68921. The cytotoxic effect of MRT68921 in the tested cancer cell lines was found to be superior to reported NUAK1 inhibitors WZ4003 and HTH-01-015. MRT68921 exerted a selective cytotoxic effect in cancer cells compared to normal cells, with an approximately 10-fold difference in IC_50_ values (Fig. 5b). The morphological changes of cancer cells after MRT68921 were observed under a microscope in Supplementary Fig. S2C. Colony formation was also significantly inhibited by low-dose MRT68921 treatment (Supplementary Fig. S2A).

### MRT68921 induces elevated ROS levels and apoptosis in cancer cells

Depletion of NUAK1 in some cancer cells induces ROS-induced cell death; likewise, elevated oxidative stress activates the NUAK1-dependent antioxidant pathway to escape from cell death. To further evaluate the effect of MRT68921 on apoptosis and ROS levels, cells treated with MRT68921 were harvested and tested by FACS analysis. As shown in Fig. 5c, the percentage of apoptotic cells (Annexin V-positive) was increased in a dose-dependent manner in NCI-H460 and MNK45 cells. Elevated ROS levels were observed in A549 cells after treatment with MRT68921 for 8 h (Fig. 5d).

### MRT68921 suppresses the NUAK1/MYPT1/Gsk3β and autophagy associated signaling pathway in cancer cells

To further evaluate the anticancer mechanism of MRT68921, the regulation of the NUAK1 and ULK1 signaling pathways was analyzed by western blot analysis. As shown in Fig. 5e, f, the cleaved PARP1 expression was remarkably increased, indicating the DNA damage of cancer cells induced by MRT68921. Previous research verified that MYPT1/Gsk3β is the critical substrate of NUAK1 kinase that protects cancer cells from oxidative stress. Western blot analysis revealed that phosphorylation of MYPT1 and Gsk3β were significantly downregulated in a dose-dependent manner after MRT68921 treatment (Fig. 5f). MRT68921 induces a slight increase of puncta LC3 in MNK45 while the tendency is not significant in U251 (Fig. 5f). To confirm whether MRT68921 could inhibit NUAK1 inhibition-induced mitophagy, we performed co-localization of mitochondria and LC3B after MRT68921 treatment for 8 h (Supplementary Fig. S3C). The co-localization phenomena were not significant, suggesting the inhibition of autophagy and mitophagy due to MRT68921 treatment. We also investigated co-localization of mitochondria and ULK1 after MRT68921 treatment compared with WZ4003 treatment, co-localization of ULK1 and mitochondria was significantly downregulated when compared with WZ4003 treatment (Supplementary Fig. S1A).

### MRT68921 inhibits cancer cell growth in a xenograft mouse model

**Fig. 6:**
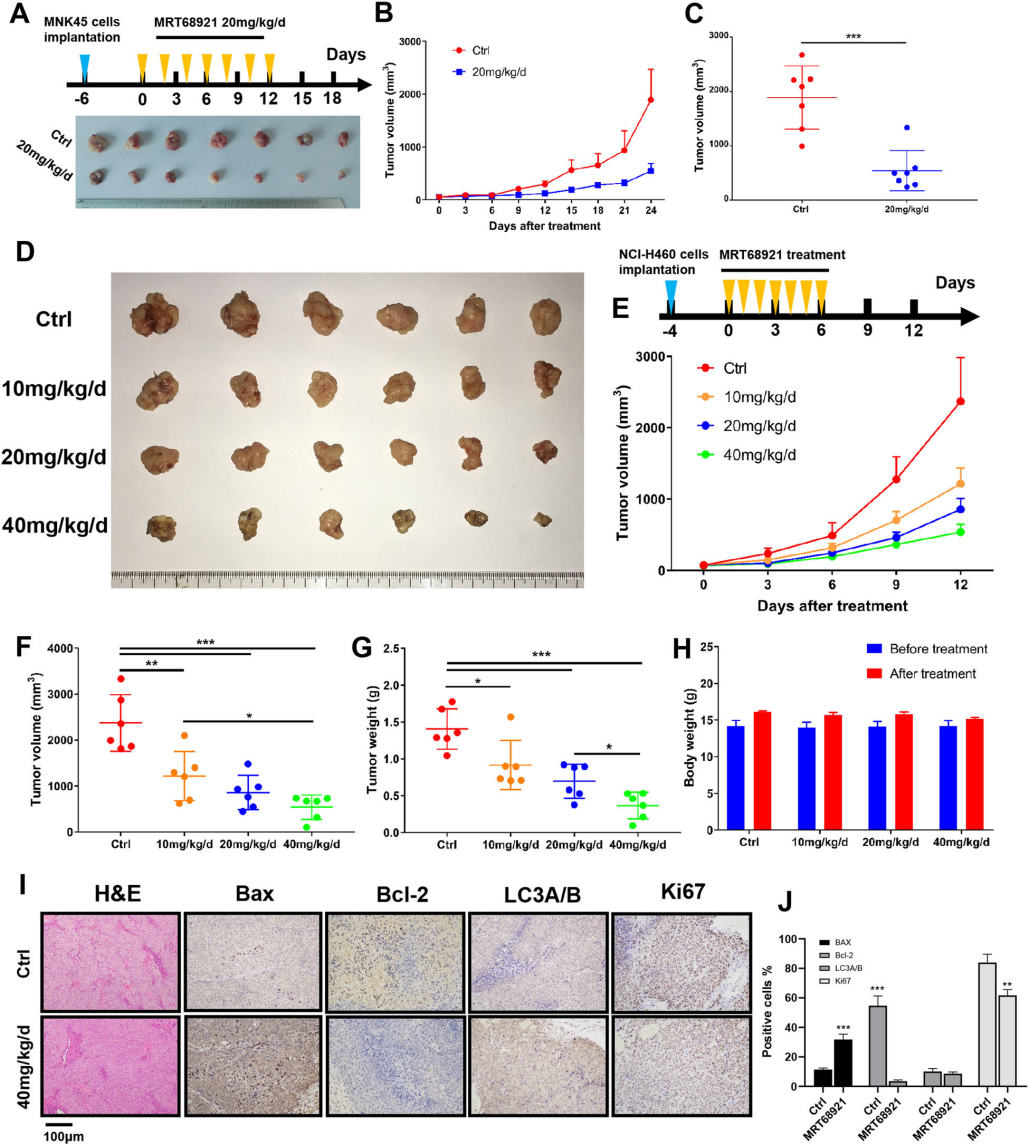
MRT68921 inhibits tumor growth in nude mouse xenograft models. **a**–**c** MNK45 cells (5 × 10^6^) were subcutaneously injected into nude mice. MRT68921 (20 mg/kg) or DMSO was injected into mice peritumorally and subcutaneously every 2 days until the seventh treatment. Tumor volumes were measured with calipers every 3 days. The tumor volumes were significantly decreased after MRT68921 treatment. Points, mean (*n* = 7); bars, standard deviation (SD). **d**, **e** NCI-H460 cells (5 × 10^6^) were subcutaneously injected into nude mice. Mice were peritumorally and subcutaneously injected with different concentrations of MRT68921 (0, 10, 20, and 40 mg/kg) every day until the seventh treatment. Tumor volumes were measured with calipers every 3 days, and body weights were monitored every 3 days. **f** At the endpoint, each treatment group had significantly decreased tumor volumes compared with the control group. The difference between the 20 mg/kg/d group and the 40 mg/kg/d group was not significant. Points, mean (*n* = 6); bars, standard deviation (SD). **g** At the endpoint, each treatment group had significantly decreased tumor weights compared with the control group. The difference between the 20 mg/kg/d group and the 40 mg/kg/d group was not statistically significant. **h** The body weights of the mice in the four treatment groups were not significantly different. **i** Immunohistochemical analysis of the expression levels of Bax, Bcl-2, LC3A/B, and Ki67 in tumor tissues (NCI-H460 xenograft). The expression of Bax increased, while the expression of Bcl-2 significantly decreased relative to the control, indicating altered apoptotic signaling. **j** Quantification results of Bax, Bcl-2, LC3A/B, and Ki67 in xenograft tumor tissues.

We next investigated the in vivo efficacy of MRT68921 using mouse xenograft models. Treatment of NCI-H460 bearing mice with MRT68921 significantly decreased the growth of tumors compared with the control group (Fig. 6d, e). The difference in final tumor volumes between the 20 mg/kg/d-treated group and the 40 mg/kg/d-treated group was not statistically significant, whereas the difference in tumor weights of these two groups was significant (*P* < 0.05, Fig. 6f, g). All treatments were well-tolerated with no significant weight loss in any of the mice (Fig. 6h). A slight ulcer was observed on the injection site in the mice of the high dose MRT68921 group (40 mg/kg/d). To examine the effect of MRT68921 on the induction of apoptosis in xenograft tumors, staining of Bax and Bcl-2 by immunohistochemistry was done in the tumors from the MRT68921 treatment group and control group. Bax expression was significantly increased while Bcl-2 expression was decreased in the treatment group compared to the control group, suggesting an apoptotic tendency induced by MRT68921 (Fig. 6i, j). The inhibitory effect of MRT68921 on tumor growth was also observed in the MNK45-bearing xenograft mouse model (Fig. 6a–c).

### MRT68921 inhibits cancer cell migration and metastasis

**Fig. 7:**
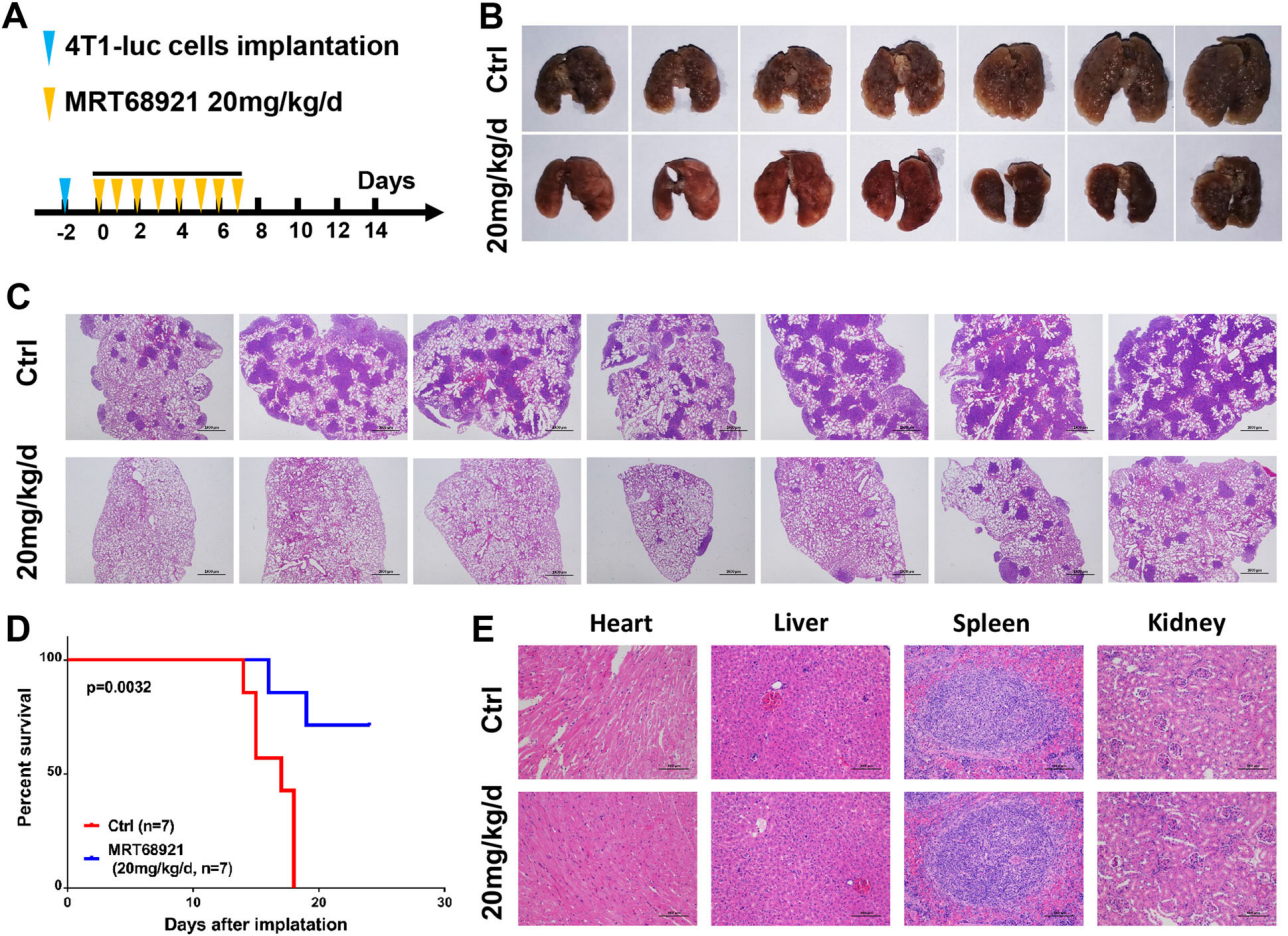
MRT68921 is efficacious in a metastatic syngeneic 4T1 murine breast cancer model. **a** 4T1 cells (2 × 10^5^) were harvested and injected intravenously into BALB/c mice. The treatment started on the third day after injection. The mice were intravenously injected with DMSO or MRT68921 (20 mg/kg/d) every day until the seventh treatment. (*n* = 7 per group). **b** Remaining mice were killed 24 days after treatment. Lungs from the vehicle- and MRT68921-treated groups demonstrated substantial differences in tumor burden. **c** H&E staining images of lungs. MRT68921 treatment significantly decreased the number of metastatic nodules, suggesting a lower tumor burden. **d** Survival curves and tumor burden of the 4T1 mice after treatment. Mice after MRT68921 treatment showed complete tumor regression and 71.4% survival over 24 days. **e** H&E staining of major organs (heart, liver, spleen, and kidney) after treatment with vehicle or MRT68921. No significant injuries were observed in either group.

Elevated NUAK1 is closely associated with cancer metastasis and patient survival via epithelial–mesenchymal transition (EMT) alteration. We observed that low-dose MRT68921 treatment (1 μM) significantly decreased wound healing rates compared with the control group in three different cancer cell lines (Supplementary Fig. S3B). MRT68921 treatment also inhibited the invasive activity of cancer cells in a transwell assay (Supplementary Fig. S3A). To further evaluate the effect of MRT68921 on cancer cell metastasis, 4T1 cell-bearing BALB/c mice were treated with MRT68921 to further evaluate its effect on cancer cell metastasis (Fig. 7a). MRT68921 treatment significantly reduced the number of lung metastatic nodules in mice (Fig. 7b). This result demonstrated that MRT68921 inhibits breast cancer metastasis to the lungs. H&E staining after paraffin sectioning further demonstrated significantly decreased metastatic nodules in the lungs of the MRT68921 treatment group (Fig. 7c). The overall survival period of mice in the MRT68921 treatment group was significantly prolonged compared to the control group (Fig. 7d). In the experiments with subcutaneous transplantation models, MRT68921 was administered peritumorally and subcutaneously, which did not represent systematic toxicity. Therefore, MRT68921 was administered intravenously, and the major organs of BALB/c mice were separated at the endpoint for H&E staining. There was no significant toxicity throughout the treatment period. Abnormal structures of the heart, kidney, liver, and spleen of each mouse were not observed (Fig. 7e).

## Discussion

The metabolic characteristics of tumors confer higher ROS production and higher levels of antioxidant proteins to maintain oxidative stress homeostasis^27^. As the amount of ROS exceeds the capacity of the antioxidant systems, oxidative stress occurs, followed by autophagy, which detoxifies ROS and relieves oxidative stress. NUAK1 is a newly proven key component of the tumor antioxidant system with huge therapeutic potential^12^. Several NUAK1 inhibitors have been reported but have not been approved for clinical applications partly because of the excessive application dosage^13,28,29^. Recently, potential chemotherapies that are targeted to the antioxidant system of tumors have been proven to be effective, but they are not sufficient to achieve acceptable clinical effects. Because protective autophagy induction followed by a deficiency of oxidative homeostasis is one of the major reasons for treatment resistance. Mitophagy is a protective mechanism of antioxidant defense therapy in cancer cells, which helps to clean the damaged mitochondria. Dual inhibition of antioxidant targets and autophagy is an applicable therapeutic approach for cancer treatment. The fact that ULK1 is the only conserved serine/threonine kinase in the autophagy cascade makes it a very attractive target for therapeutic development. The critical roles of ULK1 in promoting protective mitophagy make it potential in increasing anticancer effect of targeting oxidant stress therapy. Therefore, there remains an urgent requirement for effective and safe therapeutic agents for the treatment of tumors, and combination therapies could be an effective strategy^30^.

In this study, we proposed an effective combination strategy of dual inhibition of NUAK1 and ULK1 kinase activities, which was proved to have a synergetic cytotoxic effect on tumor cells, which promotes apoptosis and elevated levels of ROS. A strong synergetic effect was observed with a combination of WZ4003 and SBI-0206965. Co-localization results show SBI-0206965 induced blocking of ULK1-dependent protective mitophagy could be the vital role of synergistic effect. However, a weak synergetic effect was observed with the combination of WZ4003 and MRT68921. Single application of MRT68921 revealed a significant antitumor effect. To explain this phenomenon, we examined MRT68921 as a dual inhibitor of NUAK1 and ULK1 kinases and the impact of treatment on the survival and metastasis of different tumors. The NUAK1 structure was built according to the homologous template MARK4. Molecular docking showed a strong affinity between NUAK1 and known NUAK1 inhibitors or MRT68921, which is located in the same binding pocket. Thus, we suggest that MRT68921 is a potential NUAK1 inhibitor. It was confirmed that we found MRT68921 significantly downregulate the MYPT1/Gsk3β signals, direct downstream of NUAK1. Our further research work showed that MRT68921 could kill several tumor cell types by promoting apoptosis and elevated ROS levels. MRT68921 could also weaken tumor cell migration and invasion. MRT68921 could also inhibit tumor growth in xenograft models and inhibit tumor metastasis in mice. Our data demonstrate that MRT68921 treatment appears to cause lethal ROS levels not only by downregulation of NUAK1 but also by relief of the protective mitophagy triggered by ULK1.

Overall, through a series of in vitro and in vivo investigations of MRT68921, we confirmed that it possessed anticancer potential. The research of homology modeling and molecular docking provides the foundation for further development of specific NUAK1 inhibitors for the treatment of different types of cancer.

## Supplementary information

Supplementary information

Supplementary Figure 1

Supplementary Figure 2

Supplementary Figure 3

Supplementary Figure 4

## Data Availability

All data generated or analyzed during this study are included in this published article. Further details are available on request.
