# Supplementary material for: Dual targeting of NUAK1 and ULK1 using the multitargeted inhibitor MRT68921 exerts potent antitumor activities
Source: Cell Death Dis. 2020 Sep 1;11(8):712. doi: 10.1038/s41419-020-02885-0 (PMC7463258; doi:10.1038/s41419-020-02885-0)
Supplement: Supplementary file 1 — Supplementary information [file 41419_2020_2885_MOESM1_ESM.docx]

**Supplementary information**

**Fig. S1** **A**, Representative and merge images ULK1 fluorescence (green), mitotracker fluorescence (red) and DAPI fluorescence (blue) in U251 cell after treatment of WZ4003 (20μM) for 8 h. **B,** NUAK1 was knocked down by siRNA in U251 cells, and cells were cultured for 48 hours and analyzed by Western blot. NUAK1 depletion induces puncta of LC3 and downregulation of p62, indicating the activation of autophagy. **C**, Quantification results of of NUAK1, LC3B and p62 after siRNA interference.

**Fig. S2** **A,** Colony formation was also significantly inhibited by low-dose MRT68921 treatment. **B,** Morphology change of cancer cells under a microscope after 24 hours of treatment. **C,** Images under microscope represent morphology of several cancer cells treated with MRT68921 at different concentrations (0, 5 and 10 μM) for 24 h.

**Fig. S3 A**, MRT68921 treatment inhibited the invasive activity of cancer cells in transwell assays. **B**, Low-dose MRT68921 treatment (1 μM) significantly decreased wound healing rates compared with the control group in three different cancer cell lines. **C**, Representative and merge images LC3B fluorescence (green), mitotracker fluorescence (red) and DAPI fluorescence (blue) in U251 cell after treatment of MRT68921 (5 μM) for 8 h。

**Fig. S4.** **A**, The NUAK1 structure is basically consistent with the template structure. **B**, The overall identity of the amino acid sequence was 49%. **C**, The binding mode of WZ4003 with NUAK1. **D**, The binding mode of HTH-01-015 with NUAK1.
