# Supplementary figures and images for: Dual targeting of NUAK1 and ULK1 using the multitargeted inhibitor MRT68921 exerts potent antitumor activities
Source: Cell Death Dis. 2020 Sep 1;11(8):712. doi: 10.1038/s41419-020-02885-0 (PMC7463258; doi:10.1038/s41419-020-02885-0)

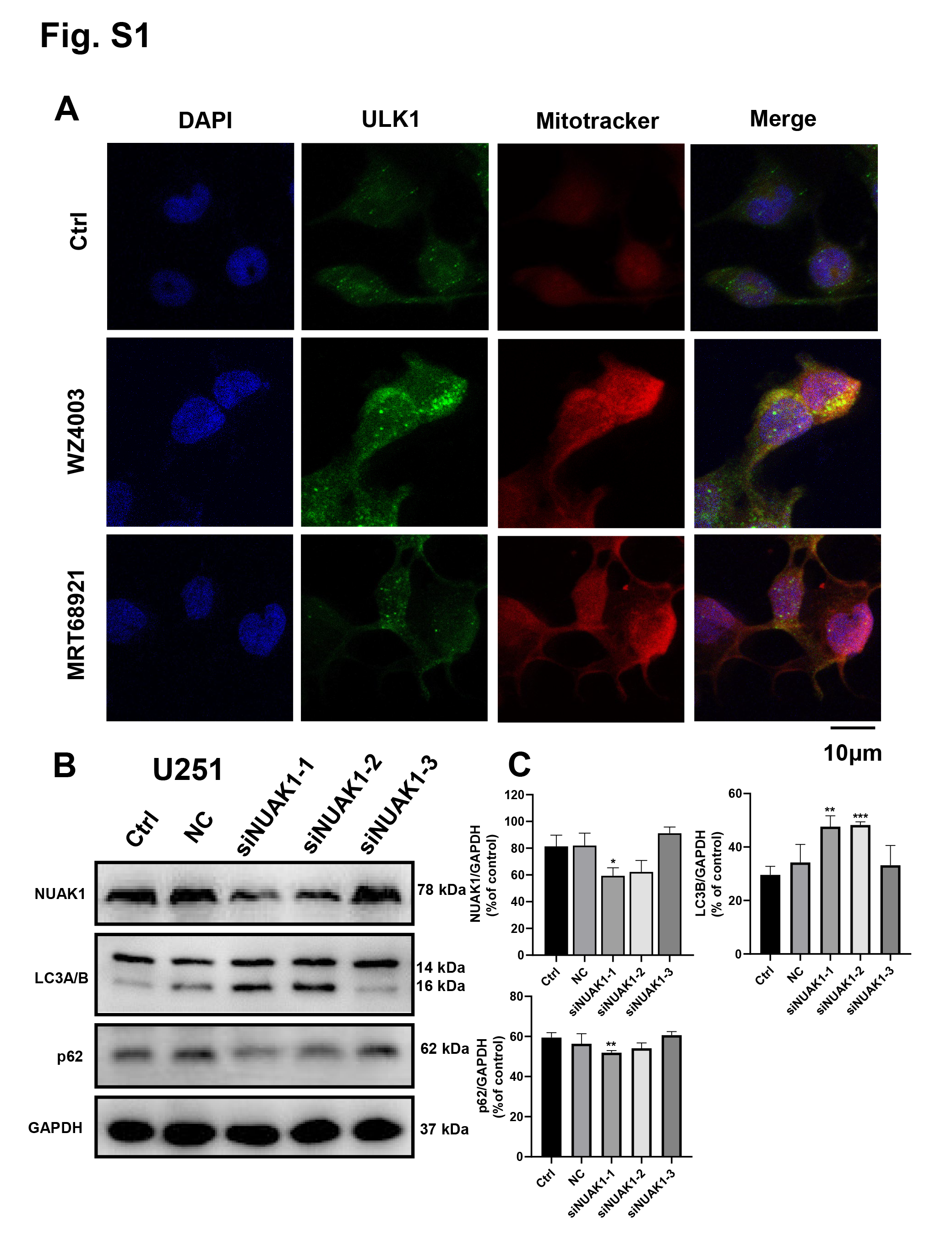

Supplement: Supplementary file 2 — Supplementary Figure 1 [file 41419_2020_2885_MOESM2_ESM.tif]

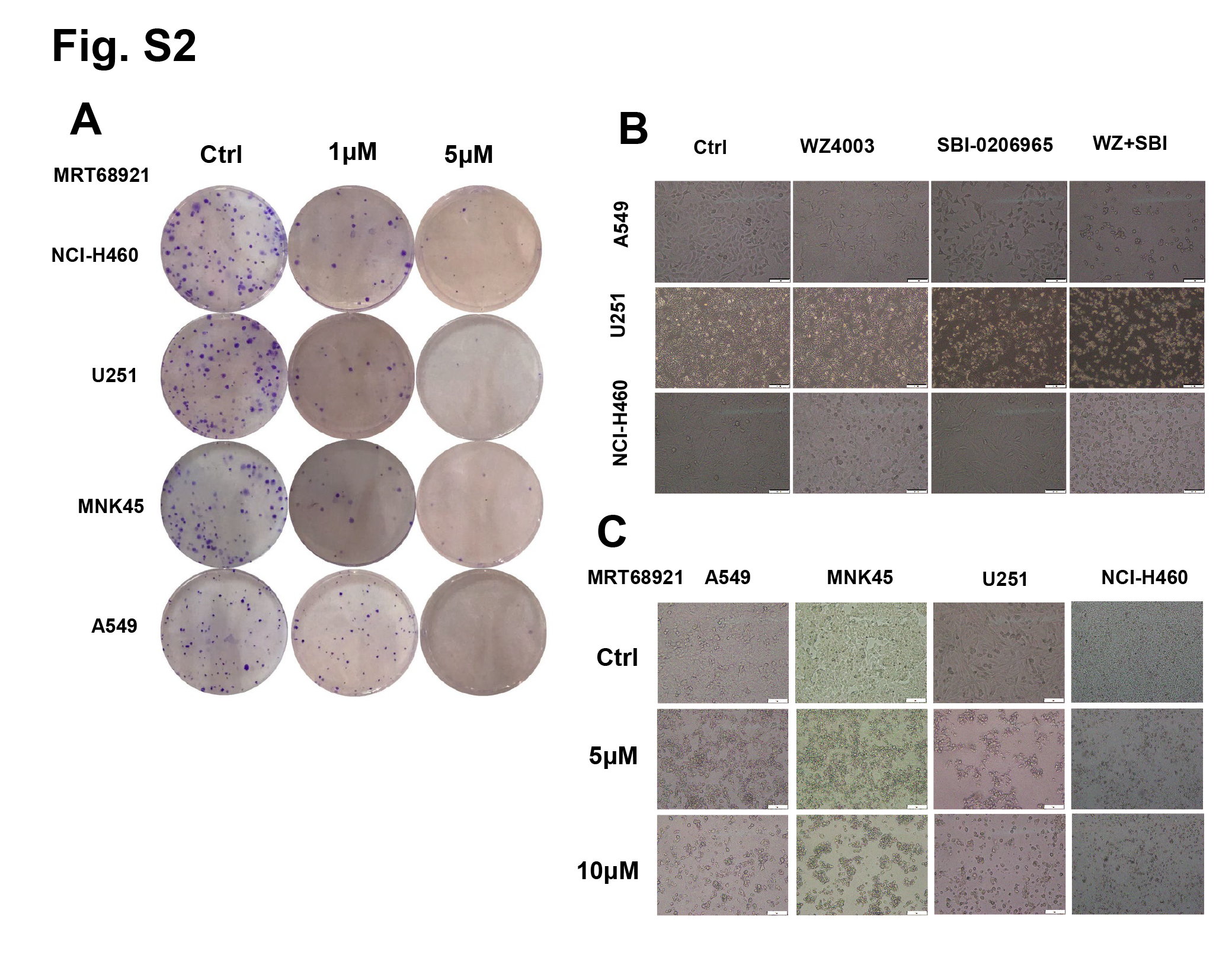

Supplement: Supplementary file 3 — Supplementary Figure 2 [file 41419_2020_2885_MOESM3_ESM.tif]

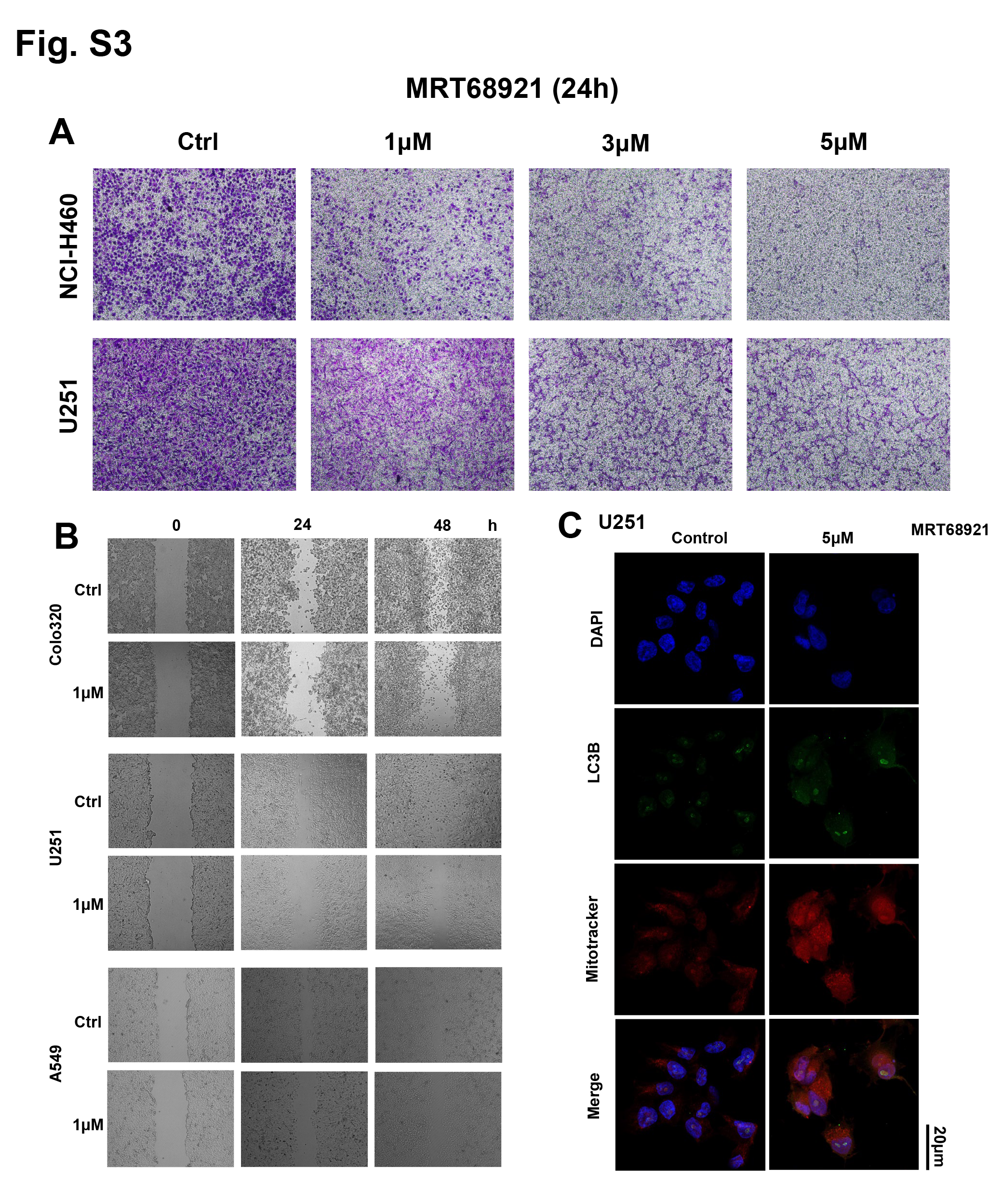

Supplement: Supplementary file 4 — Supplementary Figure 3 [file 41419_2020_2885_MOESM4_ESM.tif]

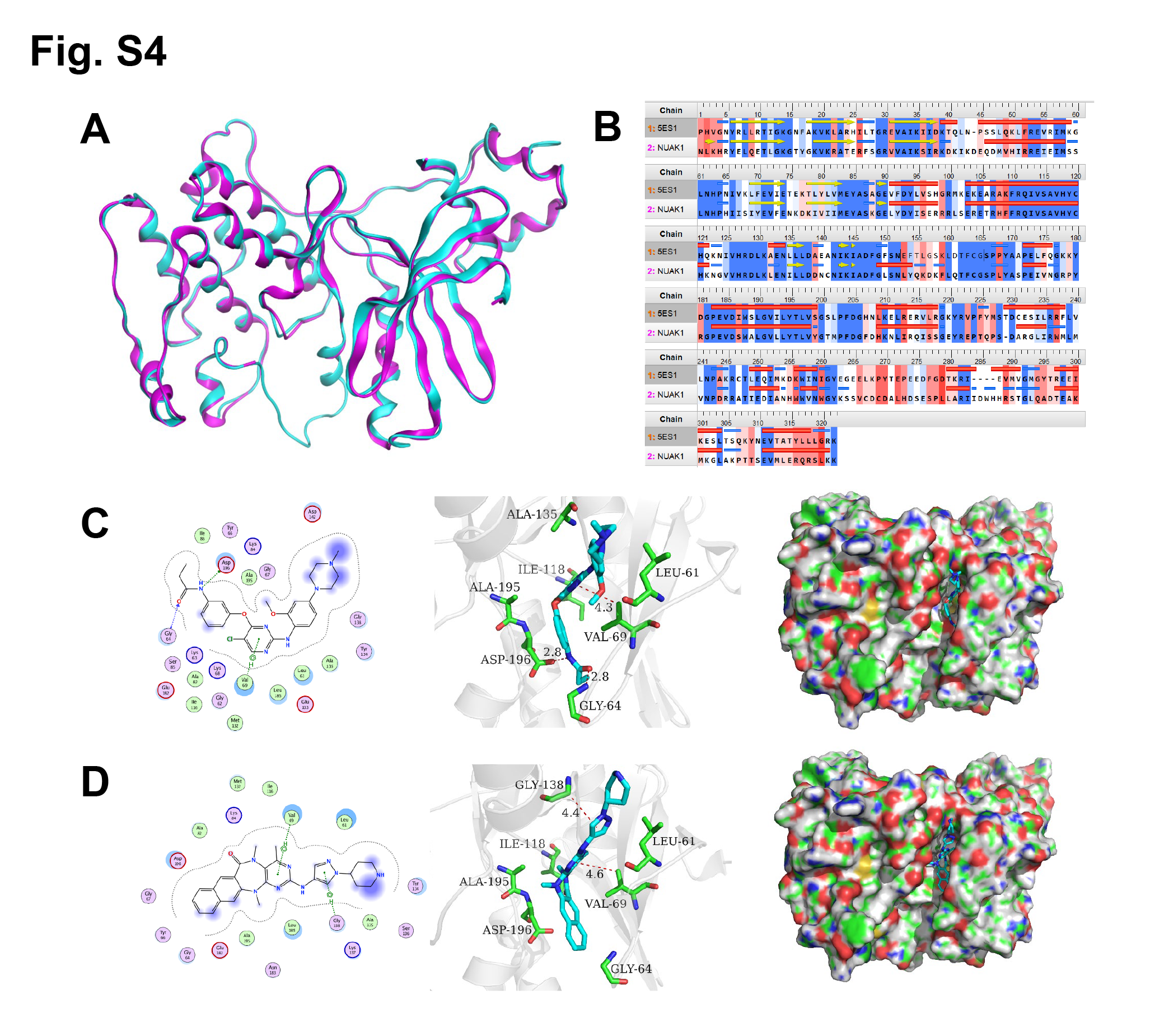

Supplement: Supplementary file 5 — Supplementary Figure 4 [file 41419_2020_2885_MOESM5_ESM.tif]
